# Tissue Regeneration with Hydrogel Encapsulation: A Review of Developments in Plants and Animals

**DOI:** 10.34133/2021/9890319

**Published:** 2021-12-02

**Authors:** Srikumar Krishnamoorthy, Michael F. Schwartz, Lisa Van den Broeck, Aitch Hunt, Timothy J. Horn, Rosangela Sozzani

**Affiliations:** ^1^Plant and Microbial Biology Department, North Carolina State University, Raleigh, NC 27695, USA; ^2^Mechanical and Aerospace Engineering Department, North Carolina State University, Raleigh, NC 27695, USA

## Abstract

Hydrogel encapsulation has been widely utilized in the study of fundamental cellular mechanisms and has been shown to provide a better representation of the complex *in vivo* microenvironment in natural biological conditions of mammalian cells. In this review, we provide a background into the adoption of hydrogel encapsulation methods in the study of mammalian cells, highlight some key findings that may aid with the adoption of similar methods for the study of plant cells, including the potential challenges and considerations, and discuss key findings of studies that have utilized these methods in plant sciences.

## 1. Introduction

Cellular potency is vastly different between plant and animal cells, with plant cells exhibiting a much higher level of regenerative capability. For instance, a large number of plant cell types exhibit pluripotency and totipotency, with stem cell niches (meristems) occurring throughout the plant body [1]. The various plant meristems consist of the apical meristems, of which the shoot and root apical meristem (SAM and RAM) are located at the tip of the shoot and root, respectively, axillary meristem (AM) in the leaf axils, and the procambium, which produces the primary vasculature within plants [2]. Generally, cells derived from almost every plant organ are capable of undergoing differentiation into multiple cell types and thus show significant regenerative capabilities, as opposed to animal cells, which show limited regenerative capabilities varying highly across different organs and species [3, 4]. Moreover, de novo generation of root and shoot meristems has been demonstrated by externally supplying varying amounts of the plant hormones auxin and cytokinin to plant cell culture [3]. This is in contrast to animals, where postembryonic cells are grouped in stem cell niches that occur in a much more localized manner within specific tissues such as the bone marrow, for the sole objective of maintaining and regenerating those specific tissues, and are unable to differentiate into other cell types. These cells are typically multipotent, where they can differentiate into multiple specific cell types that are present in a specific tissue type [5]. Moreover, fully grown animals typically lack pluripotent stem cells [6].

Conventionally, liquid suspension cultures of isolated protoplasts have been the preferred method to understand plant cell regeneration. However, despite the remarkable regenerative capacities of plant cells in nature, plant cells grown in suspension cultures have been demonstrated to have low tissue regeneration efficiency due to cell aggregation, a lack of repeatability, and generally low cell proliferation [7]. In addition, even if animal cells have been shown to have limited regenerative capabilities, researchers have achieved replicable and highly efficient regeneration of animal cells by utilizing techniques such as hydrogel encapsulation [8]. Importantly, hydrogel encapsulation allows for the formation of more physiologically representative microenvironments in tissue regeneration techniques.

Hydrogel encapsulation methods with calcium alginate have been utilized in plant sciences for specific applications such as the cryopreservation of meristems and somatic embryos of *Daucus carota*, *Solanum tuberosum*, [9] and *Cannabis sativa* [10]. However, to our knowledge encapsulation for fundamental studies on cellular regeneration has been limited. In this review, we summarize cell encapsulation methods utilized for the cellular analyses of mammalian cells and as proposed methods for plant cells. Encapsulation of plant protoplasts may allow for better comprehensive understanding of the specific mechanisms behind the cellular potency of plants and pave the way for better mechanisms of replicating plant regenerative capabilities *in vitro*.

## 2. Hydrogel-Based Cell Encapsulation, Lessons Learned from Mammalian Cell Studies

For mammalian cells, the primary motivation behind the utilization of encapsulation techniques is due to the established inadequacy of conventional cell culture methods to adequately mimic the complex *in vivo* microenvironment in natural biological conditions of mammalian cells [8]. For example, monolayer cultures do not adequately represent cell-cell and cell-extracellular matrix (ECM) interactions, which are typically responsible for cellular functions such as cell differentiation, proliferation, gene expression, and protein translation and responsiveness to other stimuli [11–13]. In certain cases, cell growth in a two-dimensional (2D) environment has very different growth outcomes than when grown within hydrogel encapsulation, which may represent a more physiologically accurate environment. For example, it has been demonstrated that when human breast epithelial cells are cultured in a standard liquid culture, they express tumor-like growth, while they revert to normal growth when cultured within encapsulated hydrogel environments such as collagen [14, 15].

The utilization of hydrogel encapsulation methods on mammalian cells have resulted in a better understanding of fundamental cellular mechanisms. For instance, the utilization of hydrogel cell encapsulation has demonstrated mechanisms of DNA repair and evasion of apostasies in cancerous cell clusters [16, 17]. The utilization of tissue organoids generated by cell encapsulation has also allowed for new understanding of the interaction between individual cells and the surrounding ECM, thus allowing for a more systematic understanding of cell behavior. For instance, the encapsulation of intestinal stem cells within hydrogels of variable mechanical properties has revealed the presence of mechanosensitive pathways that may regulate cellular survival and subsequent differentiation [18].

## 3. Hydrogel-Based Cell Encapsulation, Key Differences between Mammalian and Plant Models

**Figure 1:**
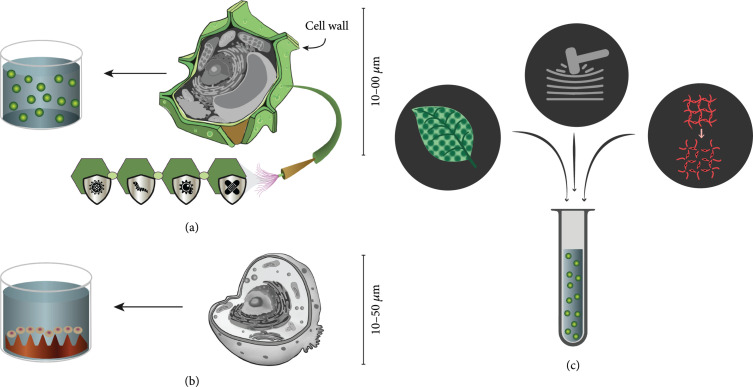
(a) Plant cells are nonadherent cells that have a cell wall which can protect against various biotic and abiotic stresses, compared to (b) mammalian cells, which are adherent cells that lack a cell wall. These differences need to be considered when (c) evaluating key properties such as mechanical strength, swelling and degradation kinetics, and material microstructure of a desired hydrogel for cell encapsulation.

The majority of mammal-derived cells are adherent cells, or anchorage dependent [19], and require their adherence to a rigid surface for nominal proliferation and survival as the absence of a culture surface can result in cellular apoptosis due to anoikis [20] and inadequate cell-matrix interactions. This indicates the need for hydrogel materials used in the encapsulation of mammalian cells to consist of the appropriate cell surface receptor-recognition motifs that are essential for cellular anchorage [21]. The majority of plant cells are anchorage independent and therefore do not undergo cellular apoptosis, which offers more flexibility for material selection in the encapsulation of plant cells. Even so, there is evidence that hydrogel matrices that more closely mimic the properties of natural plant tissue environments may yield better cell survival and proliferation [22]. On a similar note, unlike mammalian cells, plant cells comprise a cell wall, whose function is to provide structural support and protection to the cell from biotic and abiotic stresses [23], which can potentially influence the choice of encapsulation material. Protoplasts that lack cell walls due to enzymatic digestion are widely utilized in proteomic and genomic studies and studies of various aspects of plant cell physiology [7]. One key factor of protoplast maintenance to be considered is the normalization of osmotic pressure until the cell walls are fully regenerated, whereas for mammalian cells, the absence of a cell wall requires a more prolonged maintenance of osmotic pressure. Some key differences between animal cells and plant cells that may influence hydrogel encapsulation are illustrated in Figure 1.

There is a need for systematic studies that attempt to quantify effects of various gelation kinetics on the encapsulated plant cells and protoplasts. For instance, alginate is typically crosslinked using divalent cations, especially Ca^2+^. However, Ca^2+^ is also known to modulate microtubule organization and thus influence cell wall material regeneration [24], which may influence cellular response. Other factors that are considered in studies involving mammalian cell encapsulation are microstructure of the material and swelling and degradation kinetics [25], which are factors that may need to be considered in plant cell encapsulation as well.

## 4. Hydrogel-Based Cell Encapsulation, Protoplast Regeneration as Use Case for Plant Cells

A key motivation behind the utilization of hydrogel encapsulation methods in plant sciences are aimed at fundamental studies on processes associated with cell and tissue regeneration, which include the recovery of cell walls, reentry of cell cycles, formation of calli, acquisition of pluripotency, and de novo tissue regeneration [26]. Specifically, cells do not have high proliferation in liquid suspension cultures and are also not an adequate platform for studying the molecular mechanisms that influence the regeneration of cells [27]. Hydrogel encapsulation within thin alginate layers and thin alginate films (thickness<10 *μ*m) have been utilized for the immobilization of *Daucus carota* protoplasts. This allowed for the study of the effects of various culture conditions on the embryogenesis of these protoplasts [28].

**Figure 2:**
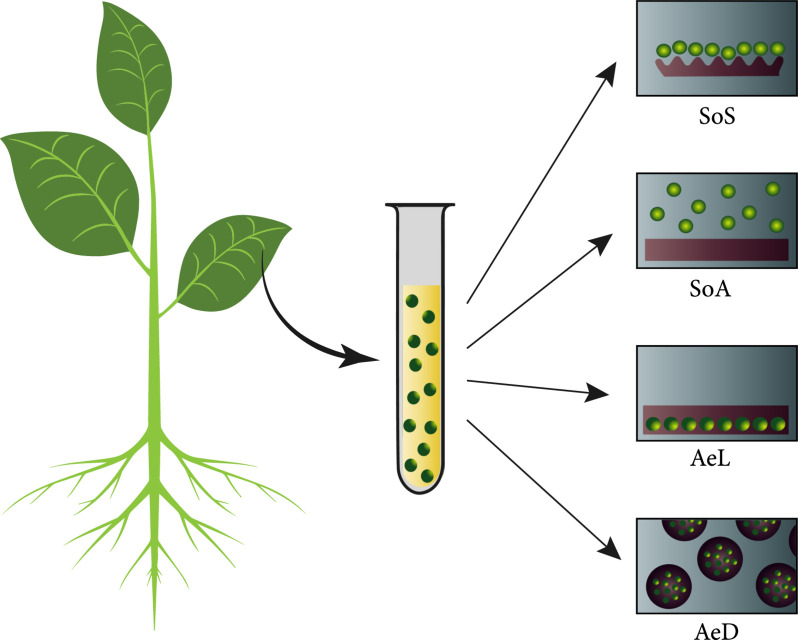
A graphical representation of the four different protoplast culture modes used by Sinha et al. [22] SoS: suspension over a rigid substrate; SoA: suspension over an agarose layer; AeL: protoplasts embedded within a single layer of agarose hydrogel; AeD: protoplasts encapsulated within agarose droplets.

Sinha and Caligari have shown the enhanced rate of protoplast division of recalcitrant lupin protoplasts when encapsulated within hydrogel droplets when compared to suspension cultures [22]. Specifically, protoplasts were isolated from cotyledons of two-week-old *Lupinus albus* seedlings and cultured in four different culture modes, as illustrated in Figure 2, to compare development in liquid suspension with hydrogel encapsulation: (i) suspension over a rigid substrate, (ii) suspension over an agarose layer, (iii) protoplasts embedded within a single layer of agarose hydrogel, and (iv) protoplasts encapsulated within agarose droplets. Subsequently, the division and the morphology of the protoplasts in the different aforementioned cases were quantified. Moreover, protoplasts encapsulated in additional hydrogels with different gelation kinetics were also compared, including different concentrations of gellan gum and sodium alginate. It was found that agarose at low concentrations were more conducive to protoplast division than other hydrogels, and it was postulated that there might be a direct relationship between hydrogel firmness and area of contact between the protoplasts and surrounding matrix. Generally, it was found that encapsulation within agarose droplets resulted in higher rates of mitosis, while alginate embedding inhibited protoplast division. It was suggested that the utilization of a continuous matrix as provided by hydrogel droplets mimics a meristematic tissue and the natural immobility of plant cells within the tissue.

Similar results were noted by Deryckere et al. when they compared three different modalities of encapsulation for plantlet regeneration of *Cichorium* protoplasts (i.e., solid media, liquid suspension, and encapsulated beads) and demonstrated enhanced regeneration in low melting point agarose beads compared to the other two modalities [29]. Specifically, the protoplasts from leaves of 11 *Cichorium* genotypes were isolated and cultured independently in the three aforementioned encapsulation methods, after which the cell viability and cell division was tracked. The cells in the liquid suspension clustered together, which in turn reduced cell viability due to the transfer of toxic compounds from one cell to another within the clusters. This was also confirmed by the detection of the build-up of anthocyanins in the liquid suspension. The fully solid agar media was impacted by the inability to be regularly refreshed, and this caused a lowering of osmotic potential due to dehydration, which lowered viability and prevented any cell division from happening. On the other hand, the cells that were encapsulated in the agar beads thrived optimally and showed the highest rates of division, due to being able to be refreshed with fresh media regularly, and the homogenous distribution of cells in the agar beads prevented cluster formation from happening. This shows that hydrogel encapsulation requires a multifaceted consideration of factors such as mode of encapsulation and may also potentially be a method to control cell-to-cell interactions, such as cluster formation.

**Figure 3:**
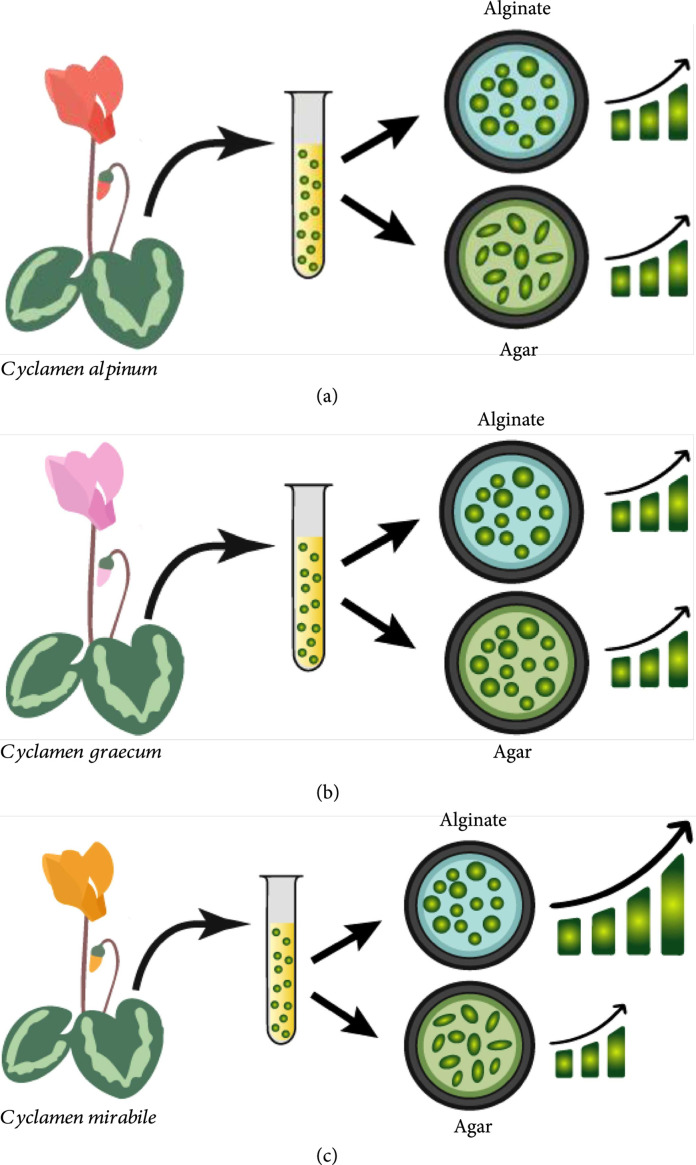
A graphical representation of the results reported by Prange et al. (bar graphs represent rates of division). (a) *Cyclamen alpinum* protoplasts showed irregular elongated morphology when encapsulated in agar and rounded morphology in alginate and showed similar rates of division in both hydrogels. (b) *Cyclamen graecum* protoplasts showed similar morphologies in agar and alginate and had similar rates of division in both hydrogels. (c) *Cyclamen mirabile* protoplasts showed irregular elongated morphology in agar and showed a much higher rate of division in alginate.

Another aspect of hydrogel encapsulation that might influence the growth of encapsulated protoplasts is the choice of hydrogel material used for encapsulation, and the genotype from which the protoplasts are isolated. For instance, Prange et al. established a regeneration protocol from protoplasts of three wild species of *Cyclamen*, *Cyclamen graecum*, *Cyclamen mirabile*, and *Cyclamen alpinum* and compared the cell growth responses in two different embedding agents, agarose and alginate, and observed different cell responses from protoplasts of different genotypes [30]. The key results from this study are illustrated in Figure 3. *Cyclamen alpinum* cells showed normal development in alginate but were elongated and bulged in agarose, with irregular cell wall strengths. However, microcalli formation was only observed after 6-10 weeks of culture in both embedding media. *Cyclamen graecum* protoplasts exhibited similar circular morphology in both alginate and agarose and showed similar rates of division and high formation of microcalli within a few days. In contrast, *Cyclamen mirabile* protoplasts showed significantly higher division rates in agarose when compared with alginate and showed irregular cell wall regeneration in agarose. This highlights the need to use a system-level approach to selecting a hydrogel material for the encapsulation of protoplasts—a specific hydrogel may elicit a different outcome in different genotypes of even the same species of plant, and it is necessary to make careful consideration of the desired outcome prior to the selection and utilization of a hydrogel material. Moreover, hydrogel selection is not a trivial choice and requires further cell-level studies to understand the effect of different hydrogels on protoplast behavior of different species and genotypes.

**Figure 4:**
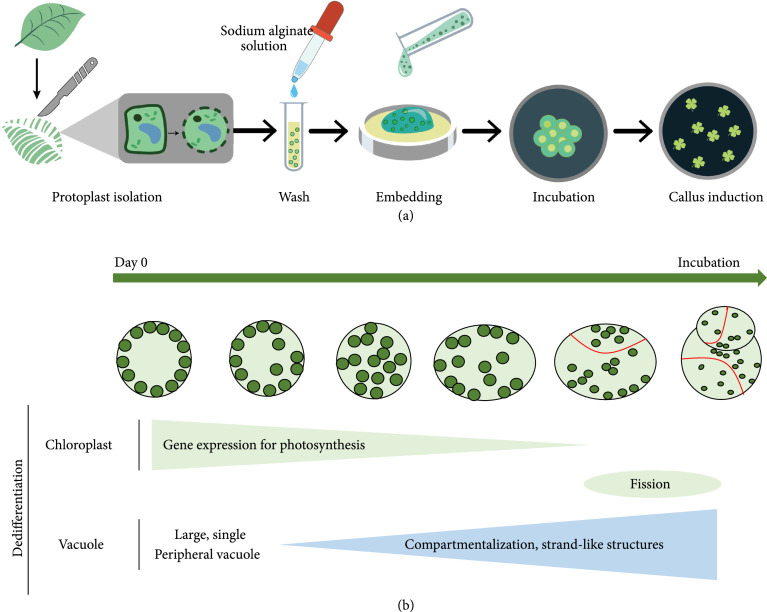
(a) Graphical representation of key steps used for isolation of protoplasts and induction of calli after alginate encapsulation. (b) Schematic of cellular dedifferentiation in protoplasts showing a downregulation of photosynthetic genes, followed by the upregulation of chloroplast genes. These are accompanied by the compartmentalization of a large single vacuole (characteristic of differentiated cells) and the appearance of strand-like structures resembling proliferating/elongating cells.

The utilization of hydrogel encapsulation methods can allow for a better in-depth understanding of protoplast behavior during processes such as division and differentiation, as shown in Figure 4. Sakamoto et al. have demonstrated the utilization of sodium alginate for encapsulating *Arabidopsis thaliana* leaf protoplasts, followed by culturing in protoplast callus growth medium (PCIM) that was supplemented with 2,4-D and thidiazuron as auxin and cytokinin, respectively [31]. Alginate encapsulation allowed for detailed analysis of the protoplast differentiation progression for better understanding of cell reprogramming and shoot regeneration. For instance, time-lapse imaging of the encapsulated protoplasts allowed for closer examination of early morphological changes and revealed that the protoplasts undergo their initial cell division between 4 and 7 days of encapsulation. Moreover, it was observed that the protoplasts undergo variable changes such as anisotropic elongation or shrinkage [31]. Changes in vacuolar morphology was tracked by time-lapse imaging of protoplasts carrying monomeric GFP- (mGFP-) tagged VACUOLAR H^+^-PYROPHOSPHATASE 1 (VHP1) (*pVHP1:VHP1-mGFP)*, and it was observed that fresh protoplasts consisted of one primary vacuole within the cell volume, followed by the appearance of strand-like structures before and during cellular elongation, followed by excessive compartmentalization of the vacuoles through successive divisions of the cells. It was discovered that the initial cell division was achieved by the biosynthesis of auxin by the activation of G2/M phase genes mediated by MYB DOMAIN PROTEIN 3-RELATED (MYB3Rs).

**Figure 5:**
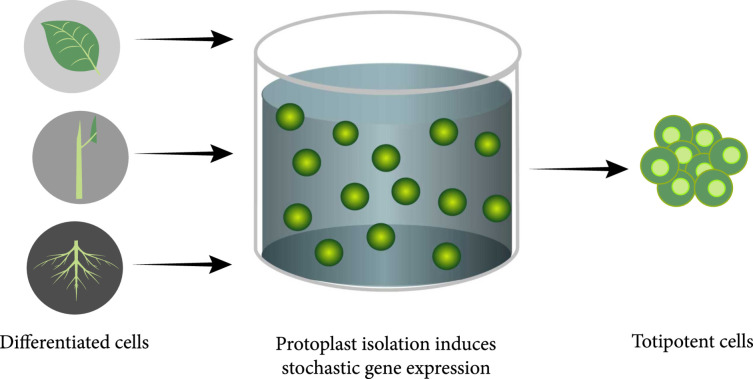
Graphical representation of the acquisition of pluripotency or totipotency in protoplasts by the digestion of cells walls due to stochastic gene expression.

The study of transcriptome changes in protoplasts may aid with the better selection of material for encapsulation. As shown in Xu et al., it demonstrated that increases in chromatin accessibility results in the stochastic activation of gene expression and enhances regeneration of protoplasts, which was visualized by the live-imaging of early protoplasts encapsulated in alginate [32]. *Arabidopsis thaliana* mesophyll protoplasts were isolated from the following transgenic lines: *pPLT7::PLT7-YFP*, *pWOX5::GFP*, *pBBM::BBM-YFP*, *pWUS::3xVenus-N7*, *pDRN::mCherry-N7*, *pDRNL::3xVenus-N7*, *pCLV3::mCherry-N7*, *pWOX2::NLS-DsRed3*, *pUBQ10::WUS-GR*, and *p35S∷DRN-GR*, and from wild-type plants, which was followed by changes to the transcriptome during regeneration of individual protoplasts. This was supplemented by the live imaging of early protoplasts by the embedding of these protoplasts on a polypropylene grid and subsequent encapsulation in alginate, followed by culturing in protoplast induction media (PIM). Long-term live imaging revealed that 0.5% of the protoplasts exhibited regeneration capabilities after 56 days of culture and formed microcalli that were greater than 200 *μ*m. To identify the specific genes that promoted totipotency acquisition, transcriptome profiles were obtained at different stages of the process, namely, immediately after protoplast isolation, and after 4, 11, 22, and 30 days of culture in PIM, and were compared with the transcriptome profiles of intact leaf cells. It was demonstrated that protoplast isolation by the digestion of cell walls tends to be a significant cause of ectopic activation of gene expression and is illustrated in Figure 5. Isolation of protoplasts tends to increase the variation in gene expression among cells, and the stochastic expression tends to endow cells with heterogeneous fates, including endowing a small portion of the cells with pluripotency or totipotency. Interestingly, the genes that were activated stochastically were compared with established gene expression profiles from different plant regeneration methods. For instance, *WUSCHEL* (*WUS*) was observed to be necessary for protoplast regeneration, with an increase in overexpression of *WUS* being correlated with increased regeneration efficiency. *PLETHORA (PLT)* genes have been previously established to be activated during regeneration due to mechanical injury to plant organs [33]. However, no PLT activation was observed during protoplast isolation, which indicated that wall digestion was the primary cause of gene expression, rather than mechanical damage to the leaves that are required prior to protoplast isolation. Additionally, prior studies have established *WUS* expression as being essential to shoot regeneration by establishing a stem cell niche [34].

## 5. Potential Challenges with Hydrogel Encapsulation Methods

While the aforementioned studies show the potential of utilizing hydrogel encapsulation as a tool for plant cell culture, there are some critical disadvantages associated with the technique that need to be considered. For instance, hydrogel encapsulation may potentially hinder downstream processes such as transcriptomic profiling due to the need to release encapsulated cells. This may require additional enzymatic degradation of the bulk matrix, such as agarases on agarose-derived hydrogels [35], or collagenases on collagen-derived hydrogels [36]. The release of encapsulated cells may also be facilitated by utilizing reversible crosslinking methods, which may allow for relatively simple liquefaction of crosslinked hydrogels. For instance, alginates are crosslinked using ionic crosslinking strategies, which are reversible by using calcium chelators such as sodium citrate [37]. Similarly, thermally crosslinked gelatin, and other related hydrogels, can be liquefied by simply varying the external temperature to the liquefaction temperature [38]. However, changes in temperature outside of an optimal window is known to cause changes in gene expression in protoplasts [39], and this must be considered before utilizing temperature-based liquefaction methods.

One of the key motivations behind using hydrogel encapsulation is being able to achieve high rates of cell division in encapsulated protoplasts and cells. However, the presence of a solid matrix may also hinder the rate of division and proliferation of some protoplasts of some species. For instance, *Chrysanthemum indicum* protoplasts have been documented to have significantly higher rates of proliferation in liquid culture when compared to hydrogel encapsulation. This may also be potentially due to accumulation of cellular waste within the hydrogel matrices [40]. Similarly, regeneration of plants from *Populus×beijingensis* was also found to be significantly higher in liquid culture, due to the ability to easily replenish fresh media. This allows for the reduction of medium osmolarity while also supplying new nutrients for the cells, which in turn can enhance division frequency [41]. Similar results were observed in the cultivation of *Kalanchoe blossfeldiana* protoplasts for plant regeneration, with the liquid culture offering a more direct approach to replenishing nutrients [42], and in the culture of leaf- and calli-derived protoplasts of *Albizia julibrissin* which showed improved division and regeneration in liquid suspension as opposed to hydrogel encapsulation in agar [43]. Thus, cases such as these may require the utilization of additional media supplantation around hydrogel constructs in order to dilute such molecules.

## 6. Conclusions

Hydrogel encapsulation is a widely utilized method in the study of cell behavior of mammalian cells that has been gaining traction in studies on plant development. More systematic studies on hydrogel encapsulation may provide new avenues for fundamental research on plant cell behavior and thus on plant development. Specifically, studies such as cell-to-cell signaling, regeneration, and organ development may be made more possible by the utilization of hydrogel encapsulation techniques on plant cells and protoplasts and may pave the way for specific applications such as improved crop development and hybridization. While there are challenges associated with encapsulation, more extensive research is required to specifically highlight the relationship between hydrogel matrix properties, and cellular behavior of encapsulated plant cells, and thus enable more fundamental plant development research by offering up pathways for the use of novel scaffolds and matrices on different species and genotypes of plants. Moreover, the utilization of hydrogel encapsulation may also be a key enabling technology to easily observe gene expression due to different cellular processes, by providing a platform for isolating and observing specific cells and tissues.

## References

[1] C. Gaillochet, and J. U. Lohmann, “The never-ending story: from pluripotency to plant developmental plasticity,” *Development*, vol. 142, no. 13, pp. 2237–2249, 2015 26130755 10.1242/dev.117614 PMC4510588

[2] M. F. Schwartz, R. Peters, A. M. Hunt, A. K. Abdul-Matin, L. van den Broeck, and R. Sozzani, “Divide and conquer: the initiation and proliferation of meristems,” *Critical Reviews in Plant Sciences*, vol. 40, no. 2, pp. 147–156, 2021

[3] K. Sugimoto, S. P. Gordon, and E. M. Meyerowitz, “Regeneration in plants and animals: dedifferentiation, transdifferentiation, or just differentiation?,” *Trends in Cell Biology*, vol. 21, no. 4, pp. 212–218, 2011 21236679 10.1016/j.tcb.2010.12.004

[4] E. W. Sinnott *Plant morphogenesis*, McGraw-Hill Book Company, 1960

[5] A. Sobhani, N. Khanlarkhani, M. Baazm, F. Mohammadzadeh, A. Najafi, S. Mehdinejadiani, and F. S. Aval, “Multipotent stem cell and current application,” *Acta Medica Iranica*, vol. 55, pp. 6–23, 2017 28188938

[6] R. Heidstra, and S. Sabatini, “Plant and animal stem cells: similar yet different,” *Nature Reviews Molecular Cell Biology*, vol. 15, no. 5, pp. 301–312, 2014 24755933 10.1038/nrm3790

[7] M. R. Davey, P. Anthony, J. B. Power, and K. C. Lowe, “Plant protoplasts: status and biotechnological perspectives,” *Biotechnology Advances*, vol. 23, no. 2, pp. 131–171, 2005 15694124 10.1016/j.biotechadv.2004.09.008

[8] K. Duval, H. Grover, L. H. Han, Y. Mou, A. F. Pegoraro, J. Fredberg, and Z. Chen, “Modeling physiological events in 2D vs. 3D cell culture,” *Physiology*, vol. 32, no. 4, pp. 266–277, 2017 28615311 10.1152/physiol.00036.2016 PMC5545611

[9] E. E. Benson, K. Harding, M. Ryan, A. Petrenko, Y. Petrenko, and B. Fuller, “Alginate encapsulation to enhance biopreservation scope and success: a multidisciplinary review of current ideas and applications in cryopreservation and non-freezing storage,” *CryoLetters*, vol. 39, no. 1, pp. 14–38, 2018 29734412

[10] H. Lata, S. Chandra, I. A. Khan, and M. A. ElSohly, “Propagation through alginate encapsulation of axillary buds of Cannabis sativa L.—an important medicinal plant,” *Physiology and Molecular Biology of Plants*, vol. 15, no. 1, pp. 79–86, 2009 23572915 10.1007/s12298-009-0008-8 PMC3550375

[11] F. Pampaloni, E. G. Reynaud, and E. H. K. Stelzer, “The third dimension bridges the gap between cell culture and live tissue,” *Nature Reviews Molecular Cell Biology*, vol. 8, no. 10, pp. 839–845, 2007 17684528 10.1038/nrm2236

[12] J. A. Hickman, R. Graeser, R. de Hoogt, S. Vidic, C. Brito, M. Gutekunst, H. van der Kuip, and IMI PREDECT consortium, “Three-dimensional models of cancer for pharmacology and cancer cell biology: capturing tumor complexity in vitro/ex vivo,” *Biotechnology Journal*, vol. 9, no. 9, pp. 1115–1128, 2014 25174503 10.1002/biot.201300492

[13] M. J. Bissell, A. Rizki, and I. S. Mian, “Tissue architecture: the ultimate regulator of breast epithelial function,” *Current Opinion in Cell Biology*, vol. 15, no. 6, pp. 753–762, 2003 14644202 10.1016/j.ceb.2003.10.016 PMC2933200

[14] O. W. Petersen, L. Ronnov-Jessen, A. R. Howlett, and M. J. Bissell, “Interaction with basement membrane serves to rapidly distinguish growth and differentiation pattern of normal and malignant human breast epithelial cells,” *Proceedings of the National Academy of Sciences of the United States of America*, vol. 89, no. 19, pp. 9064–9068, 1992 1384042 10.1073/pnas.89.19.9064 PMC50065

[15] P.-A. Vidi, M. J. Bissell, and S. A. Lelièvre, “Three-dimensional culture of human breast epithelial cells: the how and the why,” *Epithelial Cell Culture Protocols*, Springer, pp. 193–219, 2012 10.1007/978-1-62703-125-7_13 PMC3666567 23097109

[16] A. W. Holle, J. L. Young, and J. P. Spatz, “*In vitro* cancer cell -ECM interactions inform *in vivo* cancer treatment,” *Advanced Drug Delivery Reviews*, vol. 97, pp. 270–279, 2016 26485156 10.1016/j.addr.2015.10.007

[17] D. W. McMillin, J. M. Negri, and C. S. Mitsiades, “The role of tumour-stromal interactions in modifying drug response: challenges and opportunities,” *Nature Reviews Drug Discovery*, vol. 12, no. 3, pp. 217–228, 2013 23449307 10.1038/nrd3870

[18] E. A. Hushka, F. M. Yavitt, T. E. Brown, P. J. Dempsey, and K. S. Anseth, “Relaxation of extracellular matrix forces directs crypt formation and architecture in intestinal organoids,” *Advanced Healthcare Materials*, vol. 9, no. 8, p. 1901214, 2020 10.1002/adhm.201901214 PMC7274865 31957249

[19] O.-W. Merten, “Advances in cell culture: anchorage dependence,” *Philosophical Transactions of the Royal Society B: Biological Sciences*, vol. 370, no. 1661, p. 20140040, 2015 10.1098/rstb.2014.0040 PMC4275909 25533097

[20] S. M. Frisch, and R. A. Screaton, “Anoikis mechanisms,” *Current Opinion in Cell Biology*, vol. 13, no. 5, pp. 555–562, 2001 11544023 10.1016/s0955-0674(00)00251-9

[21] N. Davidenko, C. F. Schuster, D. V. Bax, R. W. Farndale, S. Hamaia, S. M. Best, and R. E. Cameron, “Evaluation of cell binding to collagen and gelatin: a study of the effect of 2D and 3D architecture and surface chemistry,” *Journal of Materials Science: Materials in Medicine*, vol. 27, pp. 1–14, 2016 27582068 10.1007/s10856-016-5763-9 PMC5007264

[22] A. Sinha, and P. D. Caligari, “Enhanced protoplast division by encapsulation in droplets: an advance towards somatic hybridisation in recalcitrant white lupin,” *Annals of Applied Biology*, vol. 146, no. 4, pp. 441–448, 2005

[23] L. Vaahtera, J. Schulz, and T. Hamann, “Cell wall integrity maintenance during plant development and interaction with the environment,” *Nature Plants*, vol. 5, no. 9, pp. 924–932, 2019 31506641 10.1038/s41477-019-0502-0

[24] R. Abdel-Basset, “Calcium/calmodulin regulated cell wall regeneration in Zea mays mesophyll protoplasts,” *Zeitschrift für Naturforschung C*, vol. 53, no. 1-2, pp. 33–38, 1998

[25] S. Krishnamoorthy, Z. Zhang, and C. Xu, “Biofabrication of three-dimensional cellular structures based on gelatin methacrylate–alginate interpenetrating network hydrogel,” *Journal of Biomaterials Applications*, vol. 33, no. 8, pp. 1105–1117, 2019 30636494 10.1177/0885328218823329

[26] Y. Y. Jeong, H. Y. Lee, S. W. Kim, Y. S. Noh, and P. J. Seo, “Optimization of protoplast regeneration in the model plant Arabidopsis thaliana,” *Plant Methods*, vol. 17, no. 1, pp. 1–16, 2021 33622383 10.1186/s13007-021-00720-x PMC7901198

[27] A. Kiełkowska, and A. Adamus, “An alginate-layer technique for culture of Brassica oleracea L. protoplasts,” *In Vitro Cellular & Developmental Biology. Plant*, vol. 48, no. 2, pp. 265–273, 2012 22593638 10.1007/s11627-012-9431-6 PMC3337407

[28] K. Maćkowska, A. Jarosz, and E. Grzebelus, “Plant regeneration from leaf-derived protoplasts within the Daucus genus: effect of different conditions in alginate embedding and phytosulfokine application,” *Plant Cell, Tissue and Organ Culture (PCTOC)*, vol. 117, no. 2, pp. 241–252, 2014

[29] D. Deryckere, T. Eeckhaut, J. van Huylenbroeck, and E. van Bockstaele, “Low melting point agarose beads as a standard method for plantlet regeneration from protoplasts within the Cichorium genus,” *Plant Cell Reports*, vol. 31, no. 12, pp. 2261–2269, 2012 22926032 10.1007/s00299-012-1335-8

[30] A. N. S. Prange, M. Bartsch, M. Serek, and T. Winkelmann, “Regeneration of different *Cyclamen* species via somatic embryogenesis from callus, suspension cultures and protoplasts,” *Scientia Horticulturae*, vol. 125, no. 3, pp. 442–450, 2010

[31] Y. Sakamoto, A. Kawamura, T. Suzuki, S. Segami, M. Maeshima, S. Polyn, L. De Veylder, and K. Sugimoto, “Transcriptional activation of auxin biosynthesis drives developmental reprogramming of differentiated cells,” *bioRxiv*, 2021 10.1093/plcell/koac218 PMC9614439 35922895

[32] M. Xu, Q. du, C. Tian, Y. Wang, and Y. Jiao, “Stochastic gene expression drives mesophyll protoplast regeneration,” *Science Advances*, vol. 7, no. 33, 2021 10.1126/sciadv.abg8466 PMC8357238 34380624

[33] M. M. Mathew, and K. Prasad, “Model systems for regeneration: Arabidopsis,” *Development*, vol. 148, no. 6, p. dev195347, 2021 33762427 10.1242/dev.195347

[34] T.-Q. Zhang, H. Lian, C. M. Zhou, L. Xu, Y. Jiao, and J. W. Wang, “A two-step model for de novo activation of Wuschel during plant shoot regeneration,” *The Plant Cell*, vol. 29, no. 5, pp. 1073–1087, 2017 28389585 10.1105/tpc.16.00863 PMC5466026

[35] M. Malmqvist, “Purification and characterization of two different agarose-degrading enzymes,” *Biochimica et Biophysica Acta (BBA)-Protein Structure*, vol. 537, no. 1, pp. 31–43, 1978 10.1016/0005-2795(78)90600-1 718980

[36] S. M. Krane, “Collagenases and collagen degradation,” *Journal of Investigative Dermatology*, vol. 79, no. 1, pp. 83–86, 1982 10.1111/1523-1747.ep12545849 6282982

[37] S. Rodriguez, H. Lau, N. Corrales, J. Heng, S. Lee, R. Stiner, M. Alexander, and J. R. T. Lakey, “Characterization of chelator-mediated recovery of pancreatic islets from barium-stabilized alginate microcapsules,” *Xenotransplantation*, vol. 27, no. 1, article e12554, 2020 10.1111/xen.12554 31495985

[38] G. Pitingolo, A. Riaud, C. Nastruzzi, and V. Taly, “Tunable and reversible gelatin-based bonding for microfluidic cell culture,” *Advanced Engineering Materials*, vol. 21, no. 8, p. 1900145, 2019

[39] Y. Zhu, W. Qian, and J. Hua, “Temperature modulates plant defense responses through NB-LRR proteins,” *PLoS Pathogens*, vol. 6, no. 4, article e1000844, 2010 10.1371/journal.ppat.1000844 PMC2848567 20368979

[40] T. Eeckhaut, and J. van Huylenbroeck, “Development of an optimal culture system for callogenesis of Chrysanthemum indicum protoplasts,” *Acta Physiologiae Plantarum*, vol. 33, no. 4, pp. 1547–1551, 2011

[41] X. Cai, and X. Y. Kang, “Plant regeneration from cell suspension-derived protoplasts of Populus × beijingensis,” *In Vitro Cellular & Developmental Biology-Plant*, vol. 50, no. 1, pp. 92–98, 2014

[42] L. Castelblanque, B. García-Sogo, B. Pineda, and V. Moreno, “Efficient plant regeneration from protoplasts of Kalanchoe blossfeldiana via organogenesis,” *Plant Cell, Tissue and Organ Culture (PCTOC)*, vol. 100, no. 1, pp. 107–112, 2010

[43] M.-S. Rahmani, P. M. Pijut, and N. Shabanian, “Protoplast isolation and genetically true-to-type plant regeneration from leaf- and callus-derived protoplasts of Albizia julibrissin,” *Plant Cell, Tissue and Organ Culture (PCTOC)*, vol. 127, no. 2, pp. 475–488, 2016

